# Women’s Autonomy and Anemia in Children under Five Years of Age: A Peruvian Population-Based Survey

**DOI:** 10.3390/nu15153436

**Published:** 2023-08-03

**Authors:** Rosa Campos-Guerrero, Xiomara Genoveva Diaz-Molina, Rodrigo Vargas-Fernández, Diego Azañedo

**Affiliations:** Faculty of Health Sciences, Universidad Científica del Sur, Lima 15067, Peru; 100032454@cientifica.edu.pe (R.C.-G.); 100033619@cientifica.edu.pe (X.G.D.-M.); jvargasf@cientifica.edu.pe (R.V.-F.)

**Keywords:** child nutrition disorders, personal autonomy, decision making, health surveys, Peru

## Abstract

To determine the association between women’s autonomy and the presence of childhood anemia in children under five years of age in Peru, a cross-sectional study utilizing data from the 2019 Demographic and Family Health Survey was carried out. The study employed generalized linear models with a Poisson distribution and log link function. Crude and adjusted prevalence ratios (aPR) were calculated, along with their corresponding 95% confidence intervals (CI), to assess the association of interest. A total of 15,815 women and their children under five years of age were analyzed. The prevalence of childhood anemia was 30.4% (95%CI: 29.5–31.3%), while the proportions of low, moderate and high autonomy of the mothers were 44.5%, 38.4% and 17.1%, respectively. Children under five years of age of women with a low level of autonomy were more likely to have anemia (aPR: 1.10; 95%CI: 1.00–1.21). Three out of ten children under five years of age suffer from anemia, and four out of ten mothers have a low level of autonomy. A low level of women’s autonomy was associated with a higher probability of anemia in children under 5 years of age.

## 1. Introduction

Target 2.2 of the Sustainable Development Goals aims to eradicate all forms of malnutrition by 2030, and to achieve international targets on stunting and wasting in children under 5 years of age [1]. Despite this, according to the World Health Organization, it is currently estimated that 40% of children under five years of age worldwide suffer from anemia [2]. Likewise, developing countries alone present a burden of 89% of global disability related to anemia in the general population [3]. Meanwhile, in India 2017, it was estimated that 8.3 million disability-adjusted life years were lost due to anemia in children under 5 years of age [4]. In Latin America and the Caribbean (LAC) in 2019, the prevalence of anemia in children under five was 21%, similar to the prevalence in Europe and Central Asia (20%), although much higher than in North America (7%) [5]. Likewise, in that same year in LAC, the highest prevalences were reported in Bolivia (37%) and Peru (30%), while the lowest were found in Costa Rica (19%), Argentina (19%), Cuba (18%) and Brazil (12%) [5]. This condition has a great impact on children, since it is associated with alterations in the gastrointestinal tract, immune system, thermoregulation, cognitive and motor function and physical performance, which in adulthood lead to a greater burden of disease and loss of work productivity [6,7,8].

Childhood anemia is associated with the presence of various factors [9,10,11,12,13], which can be grouped into health and hygiene factors, sociodemographic and economic factors and those related to food security, which involve adequate nutrition and nutritional supplementation. In the household, women play a fundamental role in the nutrition and care of children; therefore, within the framework of women’s human rights, women’s autonomy is the fundamental basis for making decisions related to food, finances and family health [14,15,16]. Women’s autonomy is a construct that has three dimensions that include the capacity to make decisions, control over finances, and degree of freedom of movement. Greater autonomy allows mothers to make decisions regarding the feeding and hygiene of their children and the distribution of resources for household care, thus influencing child health [16,17,18]. However, a study using data from low- and middle-income countries between 2000 and 2015 reported that only 19.9% of women had autonomy to make household decisions and 61.6% to make decisions about their health [19]. In this regard, low autonomy in decision-making by women could be related to anemia in children, through mechanisms such as low access to health services, as well as inadequate nutrition and nutritional supplementation in children [16,20].

To date, most studies have focused on assessing the association between women’s autonomy and child health status by focusing on anthropometric indicators, mortality, and the prevalence of diarrhea [21]. However, evidence regarding the association between women’s autonomy and anemia in children is scarce. The only study identified used data from the Sub-Saharan Africa Demographic and Family Health Survey (DHS) for the years 2006 to 2019, and reported that the odds of anemia decrease as women’s autonomy increases [22]. Although low female autonomy and a high prevalence of anemia are important social and public health problems in low- and middle-income countries such as in Latin America, their association has not been studied in this context.

Peru is one of the Latin American countries with the lowest female autonomy (only 19% with high autonomy) and the highest prevalence of anemia [5,23,24,25,26]. We hypothesize that women’s autonomy can be a crucial factor affecting the presence of anemia in children under 5 years of age. Therefore, the present study aimed to determine the association between women’s autonomy and the presence of childhood anemia in children under five years of age using the Demographic and Family Health Survey (ENDES—acronym in Spanish) 2019 conducted in Peru.

## 2. Materials and Methods

### 2.1. Study Design and Data Source

This cross-sectional study was based on ENDES data collected in 2019. ENDES 2019 is a survey conducted between January and December 2019 by the National Institute of Statistics and Informatics (an institution that regulates, controls and manages the statistical information of Peru) under the framework of the DHS Program, which is the format of many surveys conducted in low- and middle-income countries. Likewise, the ENDES aims to provide updated information on demographic dynamics, the health status of mothers and children under five and other health determinants such as communicable and non-communicable diseases [27]. In fact, the ENDES is composed of three questionnaires: (i) a household questionnaire, which collects information on housing characteristics (water source, sanitation, household equipment, etc.); (ii) an individual woman’s questionnaire, which collects information on sociodemographic characteristics, reproductive history, pregnancy of women aged 15 to 49 years and information on health characteristics of their children under five years; and (iii) a health questionnaire, which collects information on communicable and non-communicable diseases and their risk factors in people aged 15 years and older, and mental, oral and eye health in children under 12 years of age. In addition, the level of inference of the ENDES is at the national level, by urban/rural area and in the 25 departments of Peru [27]. The data presented in this study are openly available: https://proyectos.inei.gob.pe/microdatos/ (accessed on 18 May 2023).

### 2.2. Sampling and Data Collection

The sampling framework for the selection of the ENDES sample consists of information from the National Population Census XI and Housing Census VI of 2007 and the Household Targeting System Update 2012–2013 and updated cartographic material. The ENDES has a complex sampling method characterized by being two-stage, probabilistic, balanced, stratified and independent, at the departmental level and by urban and rural area. The primary and secondary sampling units are divided by area of residence: (i) clusters and private households in urban areas and (ii) rural census area and private households in rural areas [27]. The ENDES research unit is made up of the usual residents and the people who spent the previous night in the dwelling. For the year 2019, the collection of information was carried out through direct interviews by previously evaluated and trained staff, and the answers of the respondents were recorded on an electronic device (tablet). Further details on the ENDES methodology can be found in the technical report [27].

### 2.3. Inclusion/Exclusion Criteria

For the purposes of the present study, all children under five years whose mothers had complete information on the sociodemographic and economic characteristics that determined their level of autonomy and children who had a recorded hemoglobin value were included. Children whose mother had not spent the previous night at home and children aged six months or younger were excluded. Thus, a total of 15,815 mothers and children under five years of age were included in the present study.

### 2.4. Variables

#### 2.4.1. Outcome Measures

The dependent variable was the presence of anemia in children under five years of age. The cut-off point for the diagnosis of anemia was <11 g/dL. The definition of anemia in a child under five years of age was considered to be a hemoglobin value less than 11.0 g/dL based on the Technical Standard for the preventive management of anemia in children, adolescents, pregnant and puerperal women from 2017 [28]. Thus, the dependent variable was recoded as 1 (yes) when the hemoglobin value was less than 11 g/dL and 0 (no) when the hemoglobin value was greater than or equal to 11 g/dL. It should be noted that the value used in the present study was hemoglobin adjusted for the level of altitude of the child’s residence [29].

The determination of hemoglobin levels in the ENDES was performed under universal standards with a HemoCue^®^ hemoglobinometer model Hb 201+ previously calibrated by a trained anthropometrist on the same day as the interview [29]. To perform the procedure, the sample collection process first had to be explained to the mother of the child under five years of age and informed consent had to be obtained [29]. Sample collection in children aged 12 to 71 months was performed with the child on the mother’s or caregiver’s lap, with the anthropometrist holding the lateral area of the child’s hand and extending the arm diagonally downward. The middle finger was then punctured with a lancet to obtain a drop of sufficient quantity (10 microliters) to fill a microwell. For children under 12 months of age, the puncture was made in the heel area without squeezing or pressing, to obtain the appropriate amount of blood (10 microliters) for hemoglobin measurement in a microwell. The filled cuvette was then checked for air bubbles and placed parallel to the cuvette holder [29]. Finally, the anthropometrist recorded the result that appeared on the digital display of the hemoglobinometer on the collection card. Further details on sample collection can be found in the anemia measurement methodology report [29].

#### 2.4.2. Exposure

The variable of women’s autonomy was constructed based on four dimensions. Each of these dimensions was composed of different characteristics of the woman and were based on specific questions from the ENDES, as follows: (i) Related to decision-making, which includes economic characteristics: Who has the last word in deciding what to do with the money the husband earns [V743F], Who has the last word in large household purchases [V743B], Who has the last word in buying basic necessities [V743C]; health: Who has the last word in health care? [V743A]); and free transit: Who has the last word in visiting family or relatives? [V743D]) (ii) Attitudes towards violence that consider the reasons for justifying the violence experienced by the woman (based on battered wife explains that she leaves without telling him [V744A], battered wife explains that she neglects the children [V744B], battered wife explains that she argues with him [V744C], battered wife explains that she refuses to have sex with him [V744D], and battered wife explains that she burns food [V744E]); (iii) Socioeconomic aspects, which include characteristics such as employment (based on work in the last 12 months [V731]), and head of household (based on sex of the head of household [V151]); and (iv) Socio-cultural aspects such as educational level (based on highest level of education [V106]) and access to television, radio and newspapers (based on frequency of watching television [V159], frequency of listening to radio [V158], and frequency of reading newspaper or magazine [V157]).

After identifying the variables that make up women’s autonomy, the following recoding was carried out: (i) variables that make up the decision-making dimension were recoded as 1 when the woman had the last word in decision-making, and 0 when she did not; (ii) variables related to the justification for violence were recoded as 1 when the woman gave an affirmative answer and 0 when it was negative; (iii) economic aspects were recoded as 1 when the woman had worked in the last 12 months, and when the head of the household was a woman, and 0 when a negative answer was given about work and when the head of the household was a man; and (iv) for socio-cultural aspects, the following categorizations were made: educational level was coded as 0 = no education or only primary education, 1 = secondary education and 2 = higher education, while the variables on access to media were coded as 1 when the woman had access to television, radio and newspapers at least once a week or every day and 0 when she did not. After recoding the variables, an index was constructed with the sum of the final scores of each of the variables included, with the highest scores determining the highest level of autonomy. Finally, the constructed index was classified into tertiles to determine low, moderate and high levels of autonomy. The inclusion and recoding of the variables, the creation of an index and its division into tertiles were based on previous studies [25,30,31,32,33,34].

It should be noted that the mother’s information and the child’s hemoglobin determination were collected in a single interview conducted on the same day.

#### 2.4.3. Covariables

The inclusion of covariables was made on the basis of previous studies that had evaluated the association of interest [16,35,36,37]. Thus, the following variables were included: age of child (6–23, 24–35, 36–59), sex of child (male, female), age of mother (15–24, 25–34, 35–49), ethnicity (native, non-native), marital status (married, cohabiting), health insurance (yes, no), number of children (0, 1–3, 4–7), number of people in the household (0–4, 5 or more), area of residence (urban, rural), wealth quintile (poorest [Q1], poorer, middle, richer, richest [Q5]), natural region (Coast, Highlands, Jungle).

### 2.5. Statistical Analysis

The statistical program Stata version 17.0 (StataCorp, College Station, TX, USA) was used to merge the databases of mothers and their children and perform the analyses. In addition, the svy command was used to consider complex sampling and the ENDES weighting factor in all analyses. Population characteristics were reported with absolute and relative frequencies. In addition, a bivariate analysis was performed using the chi-square test to evaluate differences between the proportions of the variables and the dependent and independent variables. The association between the level of autonomy of the woman and the presence of anemia in children under the age of five years was evaluated using generalized linear models of the Poisson family, and log link functions and crude (PR) and adjusted prevalence ratios (aPR), along with their corresponding 95% confidence intervals (CI), were reported for covariates that had a value of *p* < 0.05 in the bivariate analysis of the dependent variable. Models were fitted for the following variables: child’s sex and age, mother’s age, mother’s ethnicity, mother’s marital status, number of children, number of people in the household, place of residence, wealth quintile, and natural region. Finally, the multicollinearity of the variables included in the adjusted model was evaluated, and this event was not found. Statistical significance was determined with a *p* value <0.05 in all analyses.

### 2.6. Ethical Considerations

The data used in this study were obtained from the secondary database of ENDES 2019, and each participant had previously signed an informed consent form. It should be noted that the ENDES databases are anonymized, thereby preventing the identification of participants. For the measurement of hemoglobin in the child, the surveyor asked the mother for informed consent, which had to be accepted by the mother in order to obtain the blood sample.

## 3. Results

### 3.1. Characteristics of the Study Population

A total of 15,815 mother–child pairs were included in the analysis. Most of the children were between 36 and 59 months old (45.2%) and were male (50.9%). Regarding the mothers of these children, 47.9% were between 25 and 34 years old, 9.2% were of native ethnicity, and 72.1% had between one and three children. Further information on the characteristics of the population included is shown in Table 1.

### 3.2. Anemia in Children under Five Years of Age

The prevalence of anemia in children under five years of age was 30.4% (CI: 29.5–31.3%) in 2019. The highest proportions of anemia were found in children who were aged 0–23 months (50.0%) and were male (32.5%). In addition, these higher proportions were found in children whose mothers were aged between 15 and 24 years (37.6%), belonged to a native ethnicity (45.8%), were cohabiting (32.3%), and had between four and seven children (39.9%). Additionally, the highest proportions of childhood anemia were found in children whose mothers had a low (35.1%) and moderate (27.5%) level of autonomy (Table 2).

### 3.3. Level of Mothers’ Autonomy

The proportions of low, moderate and high levels of autonomy were 44.5%, 38.4% and 17.1%, respectively. Regarding a low level of autonomy, the highest proportions were found in mothers aged 15 to 24 years (50.6%) who belonged to a native ethnicity (74.7%), were cohabiting (46.7%), had health insurance (45.1%) and had between four and seven children (71.8). More information about the characteristics according to the level of autonomy is presented in Table 3.

### 3.4. Association between the Level of Mother’s Autonomy and the Presence of Anemia in Children under Five Years of Age

In the crude analysis, it was found that low (PR: 1.44; 95%CI: 1.31–1.58) and moderate (PR: 1.13; 95%CI: 1.02–1.25) autonomy were associated with the presence of anemia in children under five years of age, while in the analysis adjusted for the child’s, mother’s and household characteristics, only women with a low level of autonomy had a higher probability of their children under five years of age having anemia (aPR: 1.10; 95% CI: 1.00–1.21; *p* = 0.047) (Table 4).

## 4. Discussion

The present study aimed to determine the association between women’s autonomy and the presence of anemia in children under five years of age in the Peruvian population in 2019. In this regard, it was found that three out of ten Peruvian children under five years of age had anemia at the time of the study. Likewise, in relation to women’s autonomy, it was determined that eight out of ten Peruvian women had low to moderate autonomy. In addition, children under five years of age of women with low autonomy were more likely to have anemia.

Children under five years of age have high nutritional demands and constitute a high-risk group for anemia, which is the main cause of years lived with disability in this age group [4,12,38]. In Peru, it is estimated that around 30% of children under five years of age suffered from anemia in 2019, a figure higher than the LAC average (21%) [5]. Although the figures are still high, a decreasing trend has been observed in the country in the last decade [5], with the prevalence of childhood anemia having reached 40% in 2010. This reduction is consistent with that reported by a study that identified absolute and relative reductions in the prevalence of anemia in children under 5 years of age in 29 low- and middle-income countries in the period 2000–2018 [39]. In this regard, the presence of low income is common in a large proportion of households in these countries, limiting the purchase of quality food and favoring the consumption of foods with high caloric content but low in micronutrients [40]. In Peru, characteristics related to a low socioeconomic level, as well as inadequate housing conditions and access to basic services, are associated with a greater probability of presenting childhood anemia [41,42,43]. Likewise, limited access to prenatal checkups, nutritional supplementation and anemia screening may increase the frequency of anemia in Peruvian children [41]. The absence of this type of care could hinder access to preventive anemia messages, as well as timely control and treatment of this condition, further contributing to its high frequency. On the other hand, although various strategies for the prevention of anemia have been implemented at the national level [44], it appears that they have not achieved the desired impact, probably due to the scarce monitoring and strengthening of these strategies, especially in vulnerable populations [45,46]. Furthermore, the design of these interventions does not account for the articulation of the plans of the different ministries involved, making their implementation inefficient [47], in addition to the fact that the multisectoral plan to combat anemia was intended to be valid until 2021, and to date no report has been published [44]. Therefore, there is a need to reevaluate the national anemia reduction strategies so that they are directed at the population at greatest risk and their incidence can be reduced; likewise, their continuity must be guaranteed.

The level of autonomy of the women assessed was found to be worryingly low, with 40% of women presenting low autonomy, while only 10% demonstrated a high level of autonomy. This finding is consistent with that reported in a previous study conducted in Africa in 2020, in which 47% of the women assessed showed low autonomy on a scale that combined four dimensions of autonomy: general, maternal and child health, financial, and social [16]. In addition, the same study showed that only half of the participants had autonomy in the care of their own health and that of their children [16]. A systematic review of studies conducted in low- and middle-income countries also showed that only 55% of women had the autonomy to decide about their own maternal health [48]. These similarities could be largely attributed to the gender roles established in these countries, which reduce women’s educational and work opportunities, diminishing their capacity to make decisions in the home [49]. Likewise, characteristics such as older age, higher educational level, urban residence and household income level have been identified as factors associated with women’s autonomy in low and middle-income contexts [48]. In Peru, with the support of various national institutions, the Ministry of Women and Vulnerable Populations has implemented policies and programs to promote gender equality and women’s empowerment in line with the sustainable development objectives of the United Nations [50]. It is essential that the progress of these policies be monitored at the national level to assess their impact on women’s autonomy.

As a main finding, it was found that women with a lower level of autonomy had a higher probability of having a child with anemia, which coincides with the results of studies carried out in Africa and Asia [22,51]. In contrast, evidence shows that women with a higher level of autonomy and financial stability tend to invest more in household nutrition, which translates into improved nutritional status and growth in children [16,35,36,37]. In addition, it has been observed that the dimension of autonomy in health-related decisions is a key factor in obtaining positive nutritional outcomes in children [16,36]. This is because mothers with greater autonomy have more access, understanding and awareness of information on child health and nutrition, allowing improved food security in the home [52]. On the other hand, a woman with greater autonomy in health care tends to seek timely and adequate care to prevent, detect and manage her own or her child’s health problems [14], which could represent greater probabilities of preventing childhood anemia through hemoglobin screening tests, and the adequate administration of iron and micronutrient supplements. We must acknowledge that the association reported in our study is on the borderline, which prevents us from drawing categorical conclusions about the relationship of interest. Consequently, we recommend further investigation of this association in different populations to ensure the consistency of results.

The public health implications of this research are diverse and require concrete actions. First, it is necessary to strengthen the supervisory bodies responsible for ensuring compliance with national strategies aimed at the prevention and timely, sustained and prolonged treatment of childhood anemia, mainly the Multisectoral Plan to Combat Anemia, the completion date of which was in 2021, and which, at present, has not shown the results of its evaluation [44]. In addition, the Ministry of Health should strengthen its role and provide constant epidemiological surveillance of childhood anemia, with the aim of directing strategies and raising awareness among authorities and the population about the impact of this public health problem on children [53]. Likewise, it is crucial that government entities in our country commit to the fulfillment of Sustainable Development Goals 2, “Zero Hunger”, 3, “Health and Well-Being, and 5, “Gender Equality and Women’s Empowerment”. These goals seek to guarantee food security and access to food for vulnerable populations, ensure the adequate provision of health services, and finally provide the necessary tools to achieve women’s autonomy in all areas and defend their rights [54].

The present study has limitations that should be considered when interpreting its results. In the first place, there may be the possibility of information bias, because the women surveyed may have provided erroneous information on the dimensions of women’s autonomy for fear of being listened to by their partners or family members, or to fit within socially acceptable standards. On the other hand, enumerators may have made recording errors or omissions when recording information on any of the variables of interest. However, the team that collected the data was trained to perform this task appropriately, minimizing this type of bias. On the other hand, the cross-sectional nature of the study prevents the establishment of cause-and-effect relationships Additionally, there are characteristics of the child (nutritional status, health, or birth characteristics) that have not been included as confounding variables due to their lack of availability and presence of missing values. Despite the limitations identified, there are strengths of the study that should be reported. First, internationally accepted measurement instruments were used to measure the exposure and outcome of interest. Similarly, the ENDES is supported by the DHS, which provides guidance for the application of population-based surveys worldwide. Likewise, the present study provides important evidence on the role of women’s autonomy in the anemia status of their children under 5 years of age, with information that allows inferences to be made at the population level.

## 5. Conclusions

A low level of women’s autonomy was associated with a higher probability of anemia in children under 5 years of age. It is necessary to strengthen the promotion of Peruvian women’s autonomy in order to empower them to make decisions regarding their children’s health care, and thus contribute to the reduction of anemia levels in children. It is also essential to evaluate the impact of anemia prevention and treatment interventions in the country in order to improve their effectiveness and ensure their continuity. The implementation of these strategies can contribute to the fulfillment of the Sustainable Development Goals related to Peruvian women and children.

## Figures and Tables

**Table 1:** Characteristics of the population included in this study.

Characteristics	*n*	Weighted Proportion *
Age of child (in months)		
6–23	5228	32.9
24–35	3450	21.9
36–59	7137	45.2
Sex of child		
Male	8048	50.9
Female	7767	49.1
Age of mother (in years)		
15–24	3421	21.2
25–34	7640	47.9
35–49	4754	30.9
Ethnicity		
Native	1461	9.2
Non-native	14,354	90.8
Marital status		
Married	4225	27.8
Cohabitant	11,590	72.2
Health insurance		
Yes	14,426	91.2
No	1389	8.8
Number of children		
0	4010	25.4
1–3	11,397	72.1
4–7	408	2.5
Number of people in the household		
0–4	7190	45.5
5 or more	8625	54.5
Area of residence		
Urban	11,083	70.1
Rural	4732	29.9
Wealth quintile		
Poorest (Q1)	4471	26.3
Poorer	4335	24.8
Middle	3129	19.6
Richer	2296	16.3
Richest (Q5)	1584	13.0
Natural region		
Coast	6569	52.8
Highlands	5260	28.8
Jungle	3986	18.4

* The weighting factor and sample specifications of ENDES were included.

**Table 2:** Characteristics of the population included according to the presence of anemia in the child, ENDES 2019.

Characteristics	Presence of Anemia		*p*-Value **
	No (*n* = 10,684) % * (95% CI)	Yes (*n* = 5131) % * (95% CI)	
Total	69.6 (68.7–70.5)	30.4 (29.5–31.3)	
Age of child (in months)			
6–23	49.9 (48.3–51.6)	50.0 (48.4–51.7)	<0.001
24–35	73.4 (71.7–75.2)	26.5 (24.8–28.3)	
36–59	82.1 (81.0–83.2)	17.9 (16.8–19.0)	
Sex of child			
Male	67.5 (66.2–68.8)	32.5 (31.2–33.8)	<0.001
Female	71.8 (70.5–73.0)	28.2 (27.0–29.5)	
Age of mother (in years)			
15–24	62.4 (60.4–64.3)	37.6 (35.7–39.6)	<0.001
25–34	69.7 (68.4–71.0)	30.3 (29.0–31.6)	
35–49	74.4 (72.9–76.0)	25.6 (24.0–27.2)	
Ethnicity			
Native	54.2 (50.9–57.6)	45.8 (42.4–49.1)	<0.001
Non-native	70.8 (69.8–71.7)	29.2 (28.3–30.2)	
Marital status			
Married	74.6 (72.9–76.1)	25.4 (23.9–27.1)	<0.001
Cohabitant	67.7 (66.6–68.8)	32.3 (31.2–33.4)	
Health insurance			
Yes	69.7 (68.7–70.6)	30.3 (29.4–31.3)	0.800
No	69.3 (66.2–72.2)	30.7 (27.8–33.8)	
Number of children			
0	72.7 (71.0–74.4)	27.3 (25.6–29.0)	<0.001
1–3	68.8 (67.8–69.9)	31.2 (30.1–32.2)	
4–7	60.1 (53.9–66.0)	39.9 (34.1–46.1)	
Number of people in the household			
0–4	71.0 (69.7–72.3)	29.0 (27.7–30.3)	0.007
5 or more	68.5 (67.2–69.8)	31.5 (30.2–32.8)	
Area of residence			
Urban	72.9 (71.8–73.9)	27.1 (26.1–28.2)	<0.001
Rural	61.4 (59.6–63.3)	38.6 (36.7–40.4)	
Wealth quintile			
Poorest (Q1)	60.0 (58.2–61.9)	40.0 (38.1–41.8)	<0.001
Poorer	67.4 (65.6–69.1)	32.6 (30.9–34.4)	
Middle	72.2 (70.2–74.2)	27.8 (25.8–29.8)	
Richer	75.1 (72.7–77.3)	24.9 (22.8–27.3)	
Richest (Q5)	82.4 (80.1–84.6)	17.6 (15.4–19.9)	
Natural region			
Coast	75.9 (74.6–77.2)	24.1 (22.8–25.4)	<0.001
Highlands	61.4 (59.6–63.2)	38.6 (36.8–40.4)	
Jungle	64.4 (62.5–66.3)	35.6 (33.7–37.5)	
Women’s Autonomy			
Low	64.9 (63.5–66.3)	35.1 (33.7–36.5)	<0.001
Moderate	72.5 (71.0–73.8)	27.5 (26.2–29.0)	
High	75.6 (73.5–77.6)	24.4 (22.4–26.5)	

CI: confidence interval. * The weighting factor and sample specifications of ENDES were included. ** Estimated *p*-value using the Chi-square test.

**Table 3:** Characteristics of the population included by level of maternal autonomy, ENDES 2019.

Characteristics	Women’s Autonomy			*p*-Value **
	High (*n* = 2567) % * (95% CI)	Moderate (*n* = 6021) % * (95% CI)	Low (*n* = 7227) % * (95% CI)	
Total	17.1 (16.3–18.0)	38.4 (37.3–39.5)	44.5 (43.3–45.7)	
Age of child (in months)				
6–23	16.9 (15.8–18.1)	38.9 (37.5–40.4)	44.2 (42.6–45.7)	0.617
24–35	17.5 (15.9–19.3)	38.7 (36.6–40.9)	43.8 (41.6–46.0)	
36–59	17.1 (15.8–18.6)	37.4 (35.8–39.1)	45.5 (43.7–47.2)	
Sex of child				
Male	17.0 (15.9–18.2)	39.0 (37.5–40.5)	44.0 (42.5–45.5)	0.467
Female	17.2 (16.0–18.5)	37.8 (36.3–39.2)	45.0 (43.5–46.6)	
Age of mother (in years)				
15–24	12.9 (11.3–14.7)	36.5 (34.4–38.7)	50.6 (48.3–52.9)	<0.001
25–34	17.8 (16.6–19.1)	40.6 (39.0–42.3)	41.6 (30.0–43.3)	
35–49	19.0 (17.4–20.7)	36.2 (34.2–38.2)	44.8 (42.8–46.9)	
Ethnicity				
Native	5.0 (3.6–6.9)	20.3 (17.4–23.7)	74.7 (71.0–78.0)	<0.001
Non-native	18.1 (17.1–19.0)	39.7 (38.6–40.9)	42.2 (41.0–43.5)	
Marital status				
Married	21.9 (20.0–23.9)	39.4 (37.3–41.6)	38.7 (36.6–40.9)	<0.001
Cohabitant	15.3 (14.4–16.3)	38.0 (36.7–39.3)	46.7 (45.4–48.1)	
Health insurance				
Yes	16.9 (16.0–17.9)	38.0 (36.8–39.1)	45.1 (43.9–46.4)	<0.001
No	18.8 (16.0–21.9)	42.2 (38.6–45.8)	39.0 (35.5–42.8)	
Number of children				
0	21.4 (19.5–23.5)	40.1 (38.0–42.2)	38.5 (36.4–40.6)	<0.001
1–3	16.1 (15.1–17.0)	38.2 (37.0–39.5)	45.7 (44.4–47.1)	
4–7	3.4 (1.1–10.2)	24.8 (18.9–31.7)	71.8 (64.4–78.2)	
Number of people in the household				
0–4	19.7 (18.4–21.1)	40.6 (39.0–42.2)	39.7 (38.1–41.3)	<0.001
5 or more	15.0 (13.9–16.2)	36.6 (35.1–38.1)	48.4 (46.8–50.0)	
Area of residence				
Urban	22.1 (20.9–23.3)	44.5 (43.2–45.8)	33.4 (32.1–34.8)	<0.001
Rural	4.6 (3.8–5.6)	22.9 (21.1–24.9)	72.5 (70.3–74.6)	
Wealth quintile				
Poorest (Q1)	2.9 (2.3–3.7)	19.6 (17.8–21.4)	77.5 (75.6–79.4)	<0.001
Poorer	10.6 (9.4–11.9)	40.8 (38.8–42.9)	48.6 (46.4–50.7)	
Middle	18.8 (17.1–20.7)	48.8 (46.3–51.3)	32.4 (30.1–34.8)	
Richer	28.6 (26.0–31.4)	47.5 (44.7–50.5)	23.9 (21.5–26.4)	
Richest (Q5)	41.1 (37.6–44.8)	44.6 (41.1–48.1)	14.3 (12.1–16.8)	
Natural region				
Coast	21.1 (19.7–22.6)	44.3 (42.6–46.1)	34.6 (32.8–36.4)	<0.001
Highlands	14.4 (13.1–15.8)	33.7 (31.9–35.5)	51.9 (49.8–54.1)	
Jungle	10.0 (8.9–11.3)	28.8 (26.7–30.9)	61.2 (58.8–63.6)	

CI: confidence interval. * The weighting factor and sample specifications of ENDES were included. ** Estimated *p*-value using the Chi-square test.

**Table 4:** Association between mothers’ level of autonomy and the presence of childhood anemia.

Variable	Crude		Adjusted *	
	PR (95% CI)	*p*-Value	aPR (95% CI)	*p*-Value
Autonomy				
High	Reference		Reference	
Moderate	1.13 (1.02–1.25)	0.015	1.03 (0.93–1.13)	0.567
Low	1.44 (1.31–1.58)	<0.001	1.10 (1.00–1.21)	0.047

PR: prevalence ratio. aPR: adjusted prevalence ratio. CI: confidence interval. Weighting factors and sample specifications of ENDES were included for all analysis. * Model adjusted for the following variables: sex and age of the child, age of the mother, ethnicity of the mother, marital status of the mother, number of children, number of people in the household, place of residence, wealth quintile and natural region.

## Data Availability

Not applicable.
